# Linear self-assembly and grafting of gold nanorods into arrayed micrometer-long nanowires on a silicon wafer via a combined top-down/bottom-up approach

**DOI:** 10.1371/journal.pone.0195859

**Published:** 2018-04-17

**Authors:** Elena Lestini, Codrin Andrei, Dominic Zerulla

**Affiliations:** 1 University College Dublin, School of Physics, Science Centre North, Dublin, Ireland; 2 University College Dublin, School of Mathematics and Statistics, Science Centre South, Dublin, Ireland; VIT University, INDIA

## Abstract

Macroscopically long wire-like arrangements of gold nanoparticles were obtained by controlled evaporation and partial coalescence of an aqueous colloidal solution of capped CTAB-Au nanorods onto a functionalised 3-mercaptopropyl trimethoxysilane (MPTMS) silicon substrate, using a removable, silicon wafer with a hydrophobic surface that serves as a “handrail” for the initial nanorods’ linear self-assembly. The wire-like structures display a quasi-continuous pattern by thermal annealing of the gold nanorods when the solvent (*i*.*e*. water) is evaporated at temperatures rising from 20°C to 140°C. Formation of both single and self-replicating parallel 1D-superstructures consisting of two or even three wires is observed and explained under such conditions.

## Introduction

Semiconductors and metals reduced to nano- or mesoscopic dimensions (1–100 nm) show different properties than those of the bulk. Such properties arise from the high degree of electronic confinement, which depends on both size and shape of the nanomaterial. [1–7]

In this regard, gold and/or silver nanoparticles have continuously attracted extensive attention over the last decade due to their unique optical properties. Indeed, in noble metal nanoparticles, which belong to a distinct class of metallic nanoparticles, properties arise from the excitation via resonant photons (resonant coupled light) within the particle size. This induces localised surface plasmon oscillations of the conduction band electrons, leading to strong surface bound electromagnetic fields that oscillate and decay exponentially over a distance comparable to the particle size (near-field Plasmon). Both amplitude and therefore intensity of the resonant coupled light increase by orders of magnitude. [3–6] These unique optical properties on gold and silver nanoparticles (but also other metals and recently more exotic materials) can be tuned by changing their size, shape, orientation and composition. [1–7]

Increasing efforts have been put into the development of reliable surfactant-assisted techniques for the synthesis of colloidal gold, silver and metal-blend nanorods [8–24] and nanowires. [24–32]

Radiative properties owing to plasmonic nanoparticles can be further tuned by deposition of these nanoparticles in an ordered manner on surfaces, exploiting the periodicity of such arrangements to generate additional conditions that fulfil the plasmon excitation conditions.

Further enhanced optical properties may arise from field-coupling between two or more neighbouring particles (gap modes) and from inter-particle orientation and separation. [1,5,20,21]

Therefore, plasmonic nanoparticles are considered important building blocks for the construction of highly ordered arrays with controlled morphology, [33–37] whose potential applications range from nanoelectronics [38,39] and surface Raman spectroscopy [40,41] to metal enhanced fluorescence employable in the field of high efficiency LEDs. [42] Plasmonic nanoparticles are also utilized to enhance the performance of devices such as dye sensitised solar cells by co-functionalisation of the ruthenium dye-sensitised TiO_2_ semiconductor system. [43]

Nanowires with physical dimensions (perpendicular to the long axis) smaller than the characteristic length of the physical property of interest are sometimes considered one dimensional (1D) nanostructures with unique electrical and optical properties to be exploited in the construction of mesoscopic devices. At present, there are several procedures for the synthesis of nanowires/nanoarrays that proceed via template-assisted growth of ionic precursors or via seed-mediated growth. [33–37]

Ultrathin gold nanowires with diameters of 1.8 nm were obtained by reduction of Au(I) to Au(0) in oleylamine-AuCl complexes through decomposition of the oleylamine polymeric strands, using silver nanoparticles as catalyst. It was found that the resulting nanowires were made of pure gold with an average length of 2 μm. [29,30]

However, the transfer of nanowires from solution onto a substrate in a controlled manner represents an extremely challenging task, mainly due to inter-wire aggregation. 1D and 2D architectures of gold-containing nanorods have been accomplished by means of scaffolding structures such as DNA [33,44,45] or biopolymer templates [33,46], by electric field alignment, [33,47] liquid crystal assembly, [33,48] self-assembly from microemulsion, [34,37,49] and scanning probe manipulation. [50–52] In this regard, atomic force microscopy of nanorods is a powerful but slow tool for controlling both the size of the final 1D array and the placement of nanorods on a substrate. [50]

Interest in spontaneous formation and assembly of gold nanoparticles in ordered arrays on a solid support is growing over the last decade. Lin *et al*. demonstrated that 1D, 2D and 3D ordered arrays can be obtained easily from CTAB reverse micelles of CTAB with gold nanoparticles. [34] Ordered arrays of parallel 1D gold nanoparticle wires can be formed from gold chloride surfactant-containing micro-heterogeneous systems, such as oleylamine/bis(ethylhexyl) sulfosuccinate salt/water/octane microemulsion systems, upon solvent drying onto a solid support and above the microemulsion percolation threshold. [35]

One of the most recent publications on this topic demonstrates how to produce ordered arrays when a non-aqueous microemulsion of silver nanoparticles is deposited on a substrate; in this case, the hexagonal phase of narrow cylindrical channels is obtained by micro-emulsifying ethylene glycol with isooctane and stabilising it with an anionic surfactant such as dioctyl sodium sulfosuccinate. Furthermore, this process is responsible for the self-alignment of the silver nanoparticles on the substrate, subsequent to the packing of ethylene glycol-rich cylinders in a hexagonal structure after evaporation of the organic solvent. [36]

However, so far the controlled, evaporative self-assembly of colloidal Gold nanorods (GNRDs) into superstructures onto top-down micropatterned substrates represents one of the most promising methods for the precise placement of self-assembled superstructures onto a solid substrate. [53]

The formation of linear arrays of nanoparticles by bottom-up approaches, for example by convective self-assembly (CSA) was researched extensively in recent years [54–56].

This paper describes a method that offers an alternate route by combining bottom-up and top-down approaches for the preparation of wire-like arrays of gold nanoclusters onto a functionalised MPTMS silicon substrate without the need for lithographically patterned substrates.

Formation of arrays was observed by controlled evaporation of an aqueous dispersion of CTAB-capped gold nanorods on a thiol-functionalised silicon substrate assembled with a removable siliconoxides-enriched silicon wafer, followed by an *in-situ* heat-induced coalescence of the assembled nanorods into gold nanoclusters.

In this combined bottom-up/top-down approach, colloidal aqueous solutions of CTAB-capped GNRDs were chosen as starting material due to the peculiar colloidal and plasmonic properties of the nanorods, as well as to their versatile preparation. [17]

## Materials and methods

Assembly of GNRDs on a grafted silicon wafer and growth of wires on the grafted silicon wafer from gold nanorods: A functionalised MPTMS silicon substrate assembled with a removable lateral siliconoxides-enriched silicon wafer was immersed in a 0.5 ml aqueous colloidal solution of GNRDs at 20°C for 30 minutes. The sample (assembled silicon substrate and GNRD solution) was put in a ventilated oven, at 20°C and the temperature was raised from 20°C to 140°C at a rate of 5°C per minute. After solvent evaporation, the temperature was kept at 140°C for 10 minutes in order to promote annealing of the GNRDs. [54] The sample was then allowed to cool down at 20°C for 10 minutes and subsequently washed with copious deionised water to remove all the water-soluble species not bound to the surface of the grafted functionalised silicon substrate (e.g. CTAB, AgNO_3_, sodium citrate and gold nanorods not bound to the silicon wafer). The sample was finally allowed to dry at 20°C in air. GNRDs assembled in a wire-like architecture.

Reference experiment: Immersion of the grafted silicon wafer in a colloidal aqueous solution of GNRDs (50 mL from the same stock solution used in the previous experiment) for 24 hours at 20°C. Finally, to remove CTAB and the water-soluble species gold nanorods not bound to the grafted silicon surface, the silicon substrate was washed with copious deionised water.

The following procedures were carried out according to literature: Synthesis of the GNRDs; [14, 17] oxidation of the removable silicon wafer; [57, 58] and additional manufacturing procedures and functionalisation of the silicon wafers with MPTMS. [59,60]

Sample characterization: Characterization of the macroscopic wires was carried out by X-ray photoelectron spectroscopy, XPS (ESCA). Detailed XPS analysis confirmed the presence of gold and thiols.

Contact angles measurements were carried out using DataPhysics OCA 15EC Contact Angle instrument (Filderstadt, Germany). Contact angle values were obtained as the mean ± standard deviation of three replicate measurements taken at different points on the surface of each sample. The silicon wafer treated with piranha solution exhibits a contact angle of 39° ± 2°. This result is in good agreement with the literature and confirms the hydrophilic nature of the substrate. When the silicon wafer was treated with MPTMS we found contact angles of 89° ± 4°, indicating the hydrophobic character of the substrate, probably due to imperfections in the MPTMS-SAMs.

Transmission Electron Microscopy (TEM): Characterization was carried out with a Tecnai G2-20 Twin microscope. This TEM is operable under variable accelerating voltages up to 200 kV. High contrast imaging with point resolution in the order of 0.3 nm and a TEM line resolution of 0.2 nm are achievable with this instrument. The microscope is computer-controlled and incorporates a modern digital imaging and analysis package.

GNRDs were deposited on TEM grids to assess the shape and diameter of the Au nanoparticles. In order to remove surfactant crystals within the solution, the colloidal (GNRD) aqueous solutions were μ-filtered using a Whatman PTFE syringe filter (0.45 μm pore size) prior to their deposition on a TEM grid. Water was allowed to evaporate, at room temperature, for 24 hours prior to TEM characterisation.

Scanning Electron Microscopy (SEM): Characterization of the MPTMS grafted silicon wafers was carried out via Scanning Electron Microscopy (SEM) using a FEI Quanta 3D Field emitter FEG-SEM under high vacuum conditions.

X-ray photoelectron spectroscopy (XPS): The chemical state of the samples’ surface was studied by X-ray photoelectron spectroscopy (XPS). Measurements were made on a VG Microlab 310D spectrometer with the Mg Kα X-ray source (hν = 1253.6 eV). The surveys were carried out at a constant dwell time of 250 ms, a pass energy of 50 eV and a step size of 0.5 eV, while the detailed spectra were taken at a dwell time of 500 ms, a pass energy of 27 eV and a step size of 0.1 eV.

The core-level signals were obtained at a photoelectron takeoff angle of 90° (with respect to the sample surface). All binding energies (BEs) were referenced to the C1s hydrocarbon peak at 284.6 eV. The peak fittings were performed using the dedicated XPS software suite Unifit 2012. The base pressure at which the XPS scans were carried out was 1 x 10^−9^ mbar.

## Results and discussion

**Fig 1:**
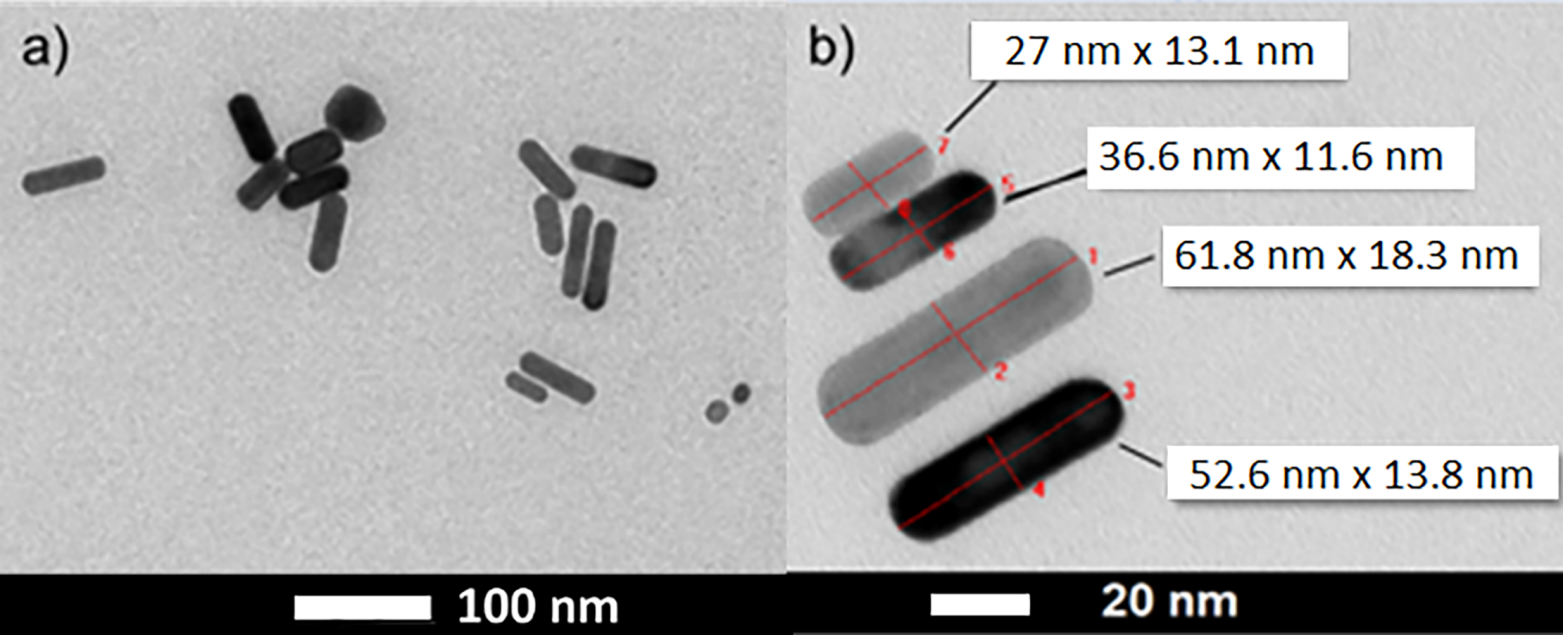
**TEM Images of Exemplary Gold Nanorods at Different Magnifications** a) as overview and b) aligned and with added physical sizes. The solution of nanorods was μ-filtered (Whatman PTFE syringe filter 0.45 μm size) prior to deposition on a TEM grid (carbon coated Cu grid).

In the here presented studies, GNRDs were used as building blocks of complex superstructures. These structures were obtained by self-assembly of GNRDs upon solvent evaporation combined with the use of a lateral siliconoxide-enriched silicon substrate. The TEM image (Fig 1) shows a typical sample of GNRDs employed in this work. The GNRDs were synthesized following successful synthetic procedures previously reported. [14,17] The nanorods were isolated after one hour from seed addition and μ-filtered in order to remove larger size crystals of CTAB formed in situ within the gold nanorods solution. No sodium sulphide (Na_2_S) was employed in the preparation in order to avoid nanoparticles aggregation [61] and Au-S bonding [62] potentially leading to Au nanoparticles with sulphur coated surfaces [63] which would, in our view, interfere with the assembly of the gold nanorods, preventing their absorption on the surface of the MPTS grafted silicon substrate.

The TEM analysis reveals selective formation of gold nanorods. The outlines of the GNRDs are as desired without dumbbell-like shapes. The physical sizes (lengths, average diameter) and therefore aspect ratios show a typical distribution centered around 40 nm x 13 nm and aspect ratios of 3–4.

**Fig 2:**
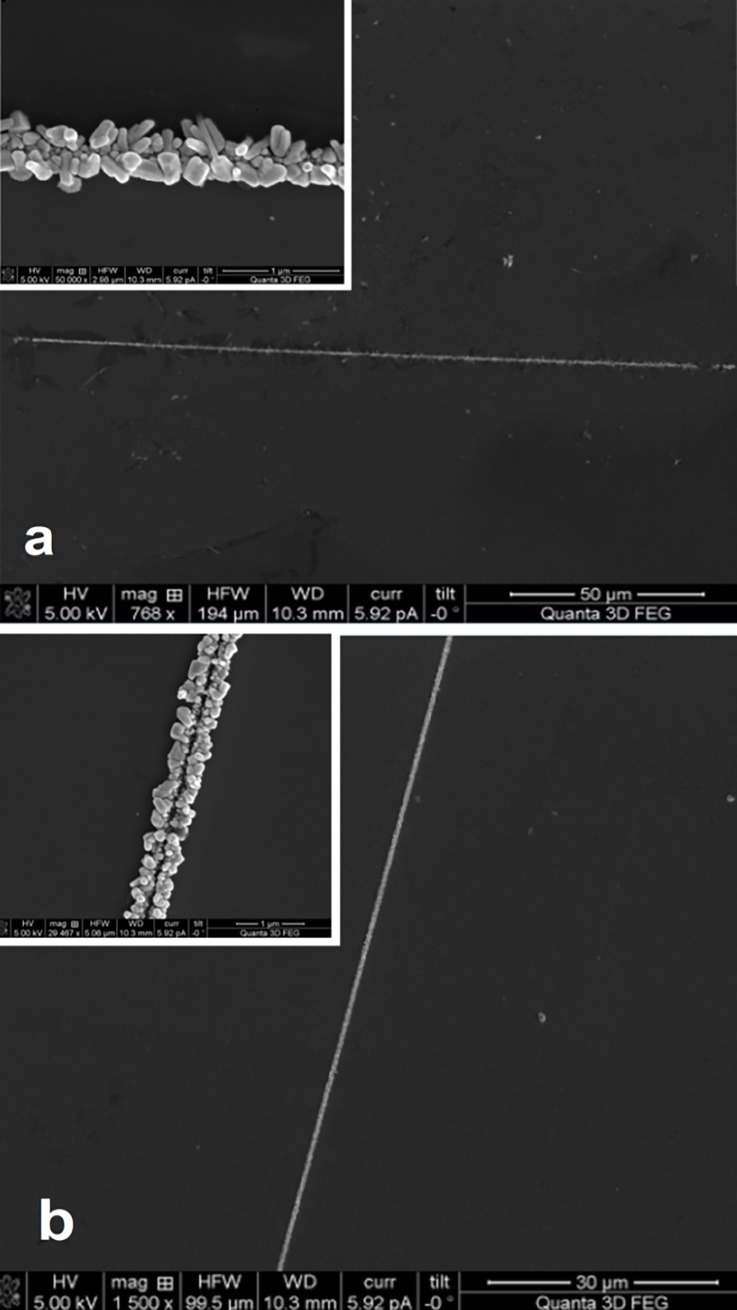
**SEM Images of Prototypes of Gold Nanowire-Like Structures** grown on MPTMS-functionalised silicon substrates by evaporation and thermal treatment of a GNRD colloidal solution in the presence of a siliconoxides-enriched silicon substrate used as “hand-rail” for the GNRDs; a) single wire with *ca*. 190 μm length, at a magnification of 768 x; b) section showing twin wires with almost 1 mm length (984.6 μm), at a magnification of 1500 x. The repeating superstructures resemble the smectic superstructures reported in literature, obtained by self-separation of GNRDs upon solvent evaporation. [64].

When the MPTS grafted silicon wafer, assembled with the removable siliconoxide-enriched silicon substrate, was immersed for 30 minutes (at 20°C) in the aqueous colloidal dispersion of gold nanorods and the resulting sample was thermally treated at temperatures raised from 20°C to 140°C (at a rate of 5°C per minute), gold nanorods assembled in wire-like superstructures. The temperature was kept at 140°C for 10 minutes in order to promote annealing of the gold nanorods in a connected-like manner. A similar procedure was previously reported in literature for thermal annealing of silver nanoparticle. [57] Detailed SEM analysis shows formation of both single and aligned twinned wire-like structures with lengths that range from a few μm up to up 1 mm (Fig 2A and 2B).

**Fig 3:**
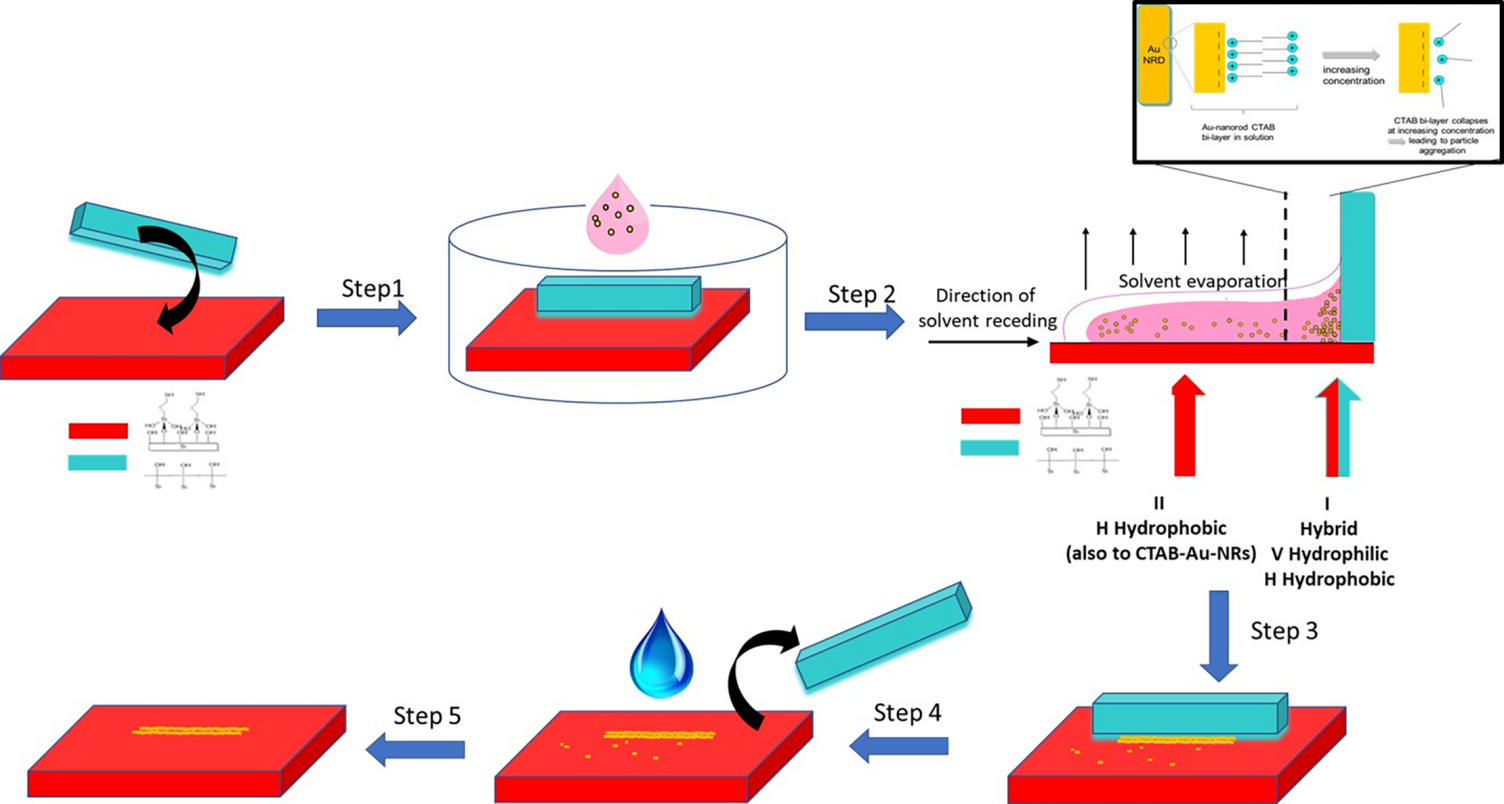
Step 1) The MPTMS-functionalised silicon wafer (red) is assembled with the oxidised silicon wafer and an aqueous colloidal solution of binary Au-NRs (solution 1 and 2; 1:1, v/v) is added (in pink in the figure); Step 2) The system is placed in an oven and the solvent is evaporated (from RT to 140°C); Step 3) The system is left in the oven for thermal annealing of the nanorods; Step 4) After cooling the silicon-wafers are disassembled and the sample is washed with deionised water and sonicated in order to remove unbound nanorods and CTAB; Step 5) The sample is dried under Nitrogen. Inset: Evaporation of the solvent and high temperature lead to CTAB bi-layer collapse and particle aggregation (close to the ruler: blue wafer in the figure). We propose that formation of wire-arrays takes place during the transition of CTAB, forming a perfect bi-layer.

Few protocols for fast CTAB-thiol exchange on CTAB-capped GNRDs have been reported in literature, [65,66] however in most cases, with similar experimental conditions as used here, it is known that ligand exchange between CTAB-capped GNRDs and thiols remains slow because of the close-packed nature of CTAB bilayer on GNRDs. [67,68] In the here presented experiments, the slow exchange of CTAB with the thiolsilane plays an important role in the formation of nanoparticle arrays. Since the CTAB-capped gold nanorods do not exchange quickly with the thiol at the MPTMS-functionalised silicon wafer, the previously uniform coverage of the MPTMS silicon substrate can be minimised. In turn, immobilisation of GNRDs on the MPTMS-functionalised silicon substrate can be promoted in specific areas of the silicon substrate by positioning a removable siliconoxides-enriched silicon substrate on the MPTMS-functionalised silicon wafer substrate (Fig 3). The hydrophilic nature of the removable substrate and the attractive interactions occurring between CTAB and the silicon oxides [69] play in our view an important role in the self-assembly and immobilization of the GNRDs in specific areas of the MPTMS-silicon substrate that are in proximity of the removable silicon wafer.

**Fig 4:**
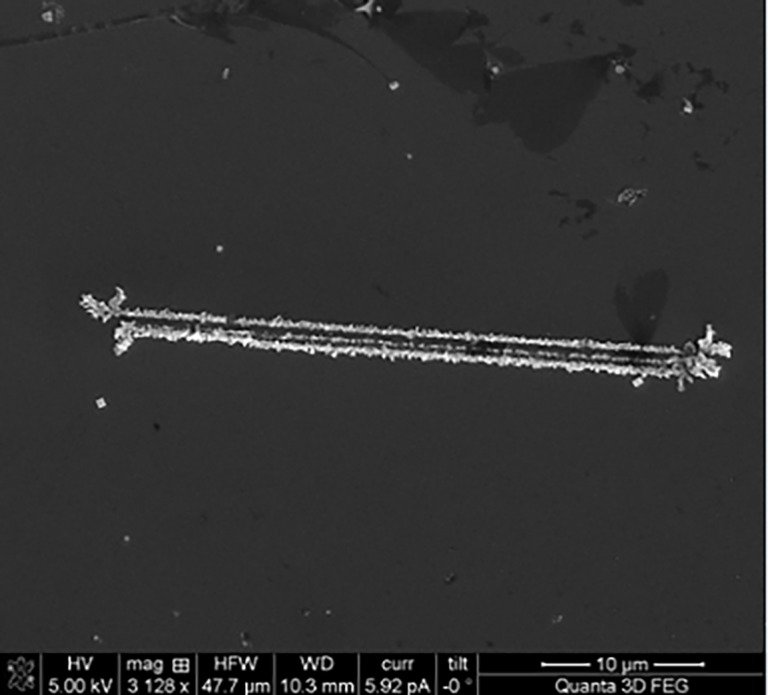
SEM image of a set of three aligned wire-like structures with ~36 μm length, at a magnification of 3128 x. The repeating superstructures resemble the smectic superstructures reported in literature, obtained by self-separation of GNRDs upon solvent evaporation. [64].

Fig 4 shows three repeating linear wires that resemble smectic superstructures, previously reported in literature [64], obtained herein by self-separation of GNRDs upon solvent evaporation.

In this work, binary mixtures of GNRDs with relative average diameters of 15±2.5 nm and 11±2 nm (Fig 1) were employed for the self-assembly with the above described MPTMS-silicon wafers. Self-separation of nanorods with relative different diameters is known to lead to the formation of their own smetic-superstructures by droplet evaporation. This behaviour, already reported in literature, occurs only upon mixing two samples of GNRDs with different relative average diameters. [64, 67] Indeed, nanorods with equal average diameter do not self-separate while those with different diameter self-separate leading to the formation of repeating superstructures (with a non-isotropic particle orientations) similar to the ones we observe in this work. However, in our arrays, no fine orientational order when compared to smectic supertructures is evident from our SEM images. In this regard, it is known that relatively fast evaporation of the solvent at temperature over 60°C, leads to the formation of less ordered superstructures as the nanoparticles cannot reach their relative equilibrium positions as they would at RT. In view of this, we propose that self-separation of nanorods with two different average-diameters still occurs leading to the formation of repeated parallel nanowires, however the orientational order of the smetic superstructure is lost due to the fast evaporation of the solvent. Moreover, GNRDs do not retain their original size but they coalesce into bigger aggregates, due to the thermal annealing process carried out at 140°C. Nevertheless, the elongated shape of these constituents is reminiscent of that of their precursors (i.e., the GNRDs), while their orientation and placement is reminiscent of the original position of the edge of the removable silicon wafer.”

**Fig 5:**
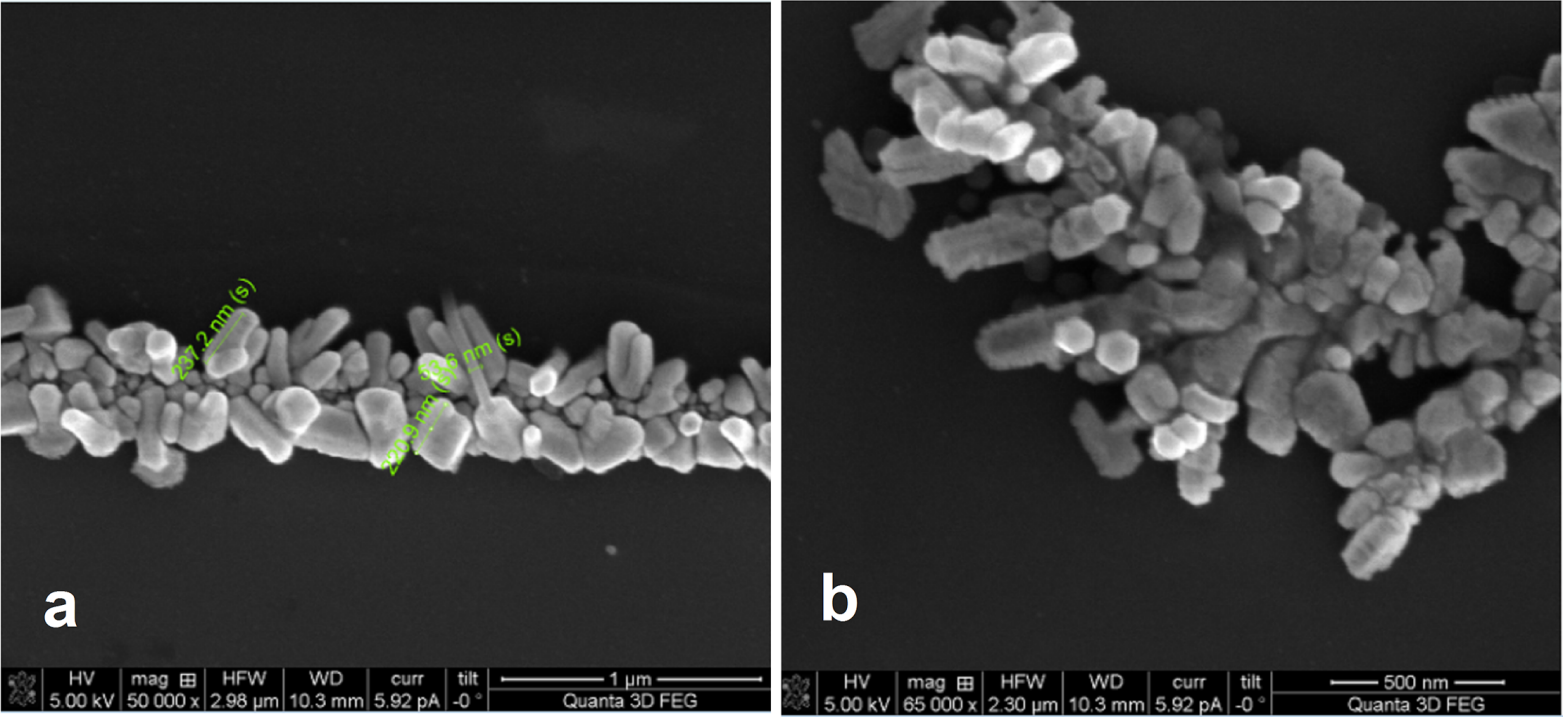
Coalescence of GNRDs into large aggregates upon thermal evaporation process.

The SEM image in Fig 5, taken at 50k x and 65k x magnification, shows the detailed inner structure of GNRDS aggregates formed upon evaporative self-assembly and the thermal process in the presence of the removable silicon support. It has been previously reported that thermal treatment of thiol-capped GNRDs can lead to coalescence of the GNRDs in ordered aggregates [70] as the thiol-alkyl chains behave more as a liquid than a solid. [71] Therefore, we propose that under such conditions the thiol-capped gold nanorods are likely to retain a certain degree of their colloidal properties and are non-isotropically distributed before being bound to the surface of a silicon substrate. This behaviour is suggested by the formation of repeating, ordered superstructures made of gold-nanorod- aggregates following evaporation of the solvent in the nanorod colloidal aqueous solution (Figs 2B (inset), 4 and 5).

The repeating superstructures are similar to those previously reported by the group of Wang and Chen, in which smectic superstructures were formed by self-separation of CTAB-gold nanorods upon solvent evaporation, [64] the difference being that in our work these superstructures are bound to the silicon substrate via S-Au bonds and that formation of single wire-like superstructures spanning microns to millimeters is also observed (e.g. Fig 2A). As a starting point one could propose two possible explanations for the self-assembly of the GNRDs in a single wire-like superstructure: Firstly, such self-assembly can be ascribed to a nematic superstructure from which the repeating superstructures enucleate from; alternatively, its formation can be attributed to the fine tuning of experimental conditions (e.g. evaporation rate, viscosity, surface tension, CTAB concentration, GNRDs average diameter) only, resulting in the formation of repeating lines and the superstructure’s self-replication. [72] Self-replication is used in the sense that structures (i.e. in our case simple straight lines) are forming in parallel to already formed similar structures without the need of additional lithographic steps. The considerable length on the order of hundreds of microns is caused by the use of the removable silicon substrate that acts as a lateral pull for the GNRDs. However, when the silicon “hand-rail” was used and solvent evaporation was carried out at 40°C, less defined superstructures were formed (S2 Fig). This indicates that slow evaporation is not the only key to the formation of the wire arrays; indeed, both temperature and attractive pull interaction between CTAB in GNRDs and siliconoxides owing to the removable silicon wafer are important to the coalescence of the nanoparticles and their precise placement onto the MPTMS-silicon substrate, respectively. Moreover, without the use of the lateral silicon-oxides-enriched substrate, no arrays were formed. Growth of the wires did neither occur when the grafted silicon wafer was immersed in the bulk solution (S3 Fig) for 24 hours nor when the gold-nanorods-containing solution was drop-casted onto the grafted silicon wafer and the solvent was evaporated at 20°C (S3 Fig). Finally, no more than three repeating wire-like superstructures were found. This indicates that the attractive interactions between siliconoxides and CTAB are not effective in promoting long-range wire self-replication (on macroscopic scales) on the timescale of the evaporation process.

**Fig 6:**
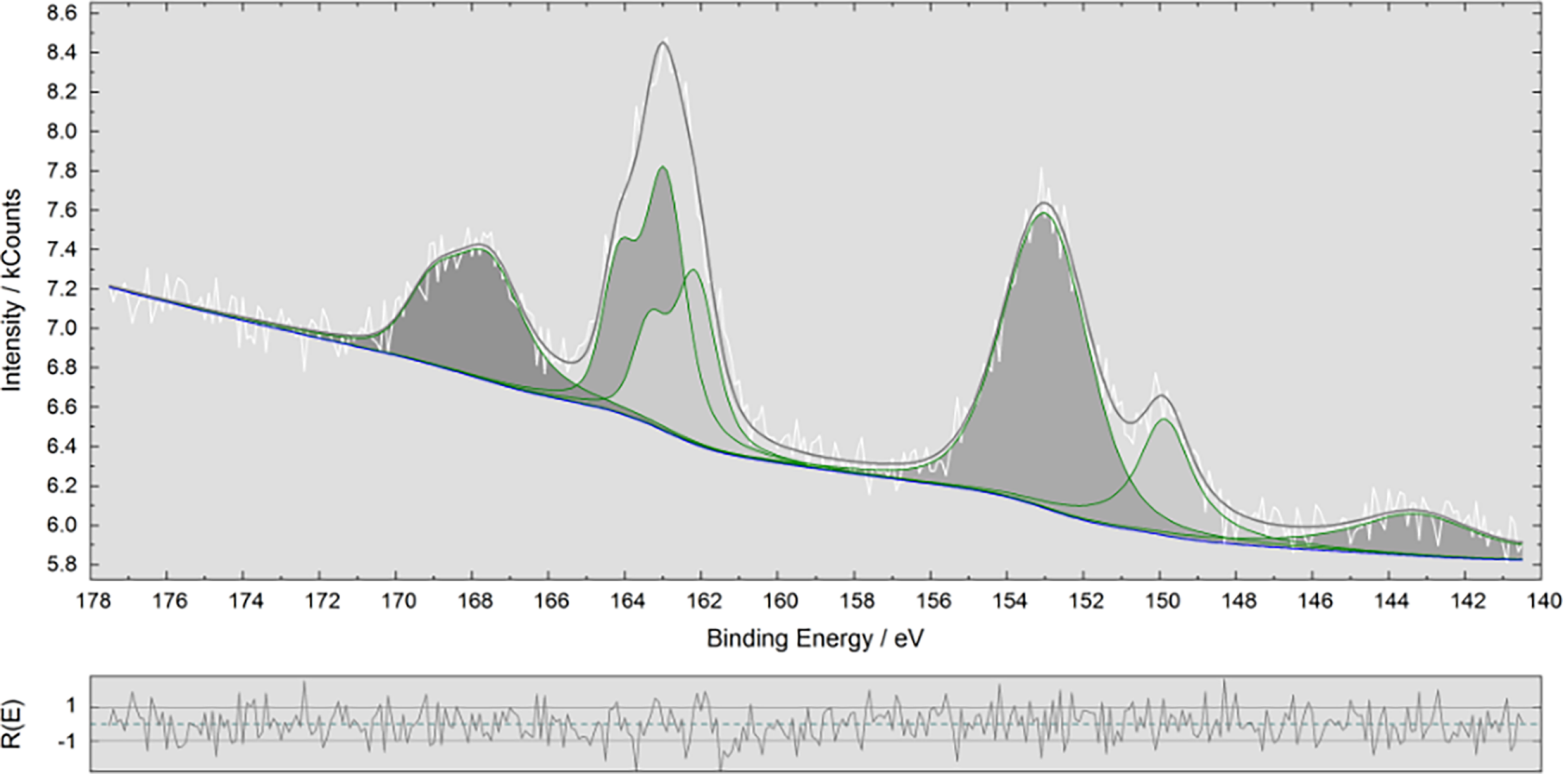
Detailed XPS analysis of the S 2p spectral region for the wire-like superstructures on MPTMS silicon wafer. The residual, indicating a reasonably well-behaved fit, is shown in the lower part.

Characterization of the macroscopic wires was carried out by XPS/ESCA spectroscopy. XPS analysis confirms the presence of gold by its strong doublet at 84.0 eV which is assigned to the core-level 4f electrons of gold atoms (S4 Fig). Moreover, sulphur species were found as demonstrated in Fig 5. A detailed analysis of the S 2p_3/2_ peak at 161.9 eV confirms the presence of sulphur which is part of the MPTMS grafted silicon wafer which provides via the thiol (Au-S) bond a potential anchor for the GNRDs (Fig 6). However, the Au bonded sulphur itself is not detectable here, as it is geometrically shadowed by the GNRDs. This finding indicates that the gold wire structures are bound to the MPTMS ad-layer through S-Au bonds. [73] However, the following peaks were also found: a peak at 163 eV (S 2p1/2 at ca. 164.36 eV), which was assigned to unbound or damaged thiol, and a peak at 167 eV (S 2p_1/2_ at 168 eV), which was assigned to oxidised species, such as sulfinates (-SO_2_-) or sulfonates (-SO_3_-). [74–76]

The latter findings are a typical result caused by radiation damage of the thiolates by the ionizing radiation (X-rays) and secondary electrons of the non-monochromatized X-ray XPS source. [74,75] However, it cannot be excluded that sulfinates could also be formed by air-oxidation of thiol-layers in the presence of polycrystalline gold. [77]

## Conclusions

We have reported on a straightforward preparation of wire-like superstructures built up from nanoscale particles with considerable macroscopic lengths obtained by evaporative self-assembly of GNRDs onto an MPTMS grafted silicon substrate and assisted by attractive interactions occurring between the siliconoxides owing to a removable silicon substrate and CTAB in CTAB-capped GNRDs. The nanorods self-assemble in microscopic linear superstructures following evaporation of the solvent at increasing temperatures leading to partial annealing of the GNRDs in the superstructures. The procedure enables the generation of single wire-like superstructures together with double and triple repeating wire-like arrays. It is proposed that formation of the repeating superstructures is driven by solvent evaporation at relatively high temperatures (above 40°C), that enables self-separation of the GNRDs before they are bound to the MPTMS-silicon substrate via S-Au bonds. The thiol linker acts more as a liquid rather than a solid at these temperatures, allowing GNRDs to retain a certain degree of their colloidal nature and self-separate forming parallel superstructures.

## Supporting information

S1 Fig Manufacturing of wire-like structures employing Si-SiO_x_-enriched removable silicon wafer. (TIF)

S2 Fig Thermal evaporation of an aqueous dispersion of GNRDs in the presence of the removable silicon wafer but carried out at 40°C on an MPTMS-functionalised silicon substrate, leads to formation of less defined super-structures. Compare with discussion in the main article.
(TIF)

S3 Fig **SEM analysis of a) product from grafted silicon wafer immersed in the bulk solution of gold nanorods for 24 hours; b) product from evaporation of drop-casted gold nanorods solution at 20°C. In absence of the Si-SiO**_**x**_ **removable silicon wafer, no arrays were formed.** Please note additionally the high density and uniformity (compared to the TEM analysis in the main article) when using a wider field of view (SEM, here 2 μm x 2 μm field of view).
(TIF)

S4 Fig XPS survey for assembled gold wires on MPTMS-grafted silicon wafer. (TIF)
