# Supplementary figures and images for: Linear self-assembly and grafting of gold nanorods into arrayed micrometer-long nanowires on a silicon wafer via a combined top-down/bottom-up approach
Source: PLoS One. 2018 Apr 17;13(4):e0195859. doi: 10.1371/journal.pone.0195859 (PMC5903609; doi:10.1371/journal.pone.0195859)

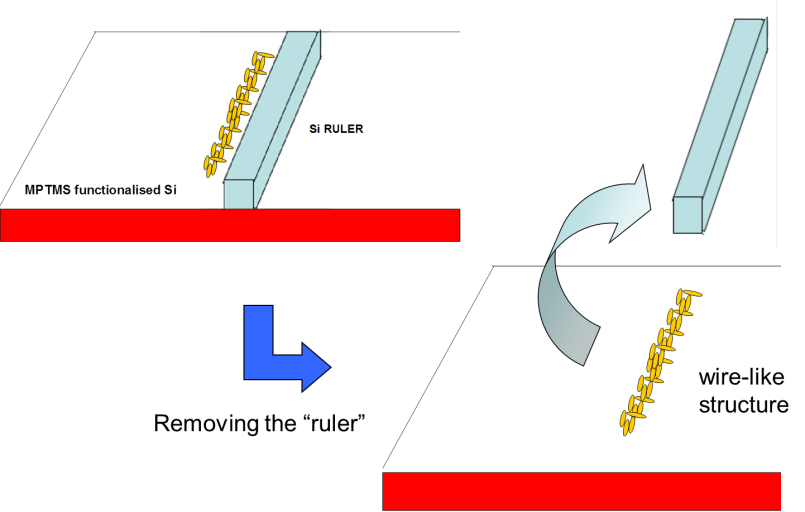

Supplement: S1 Fig — (TIF) [file pone.0195859.s001.tif]

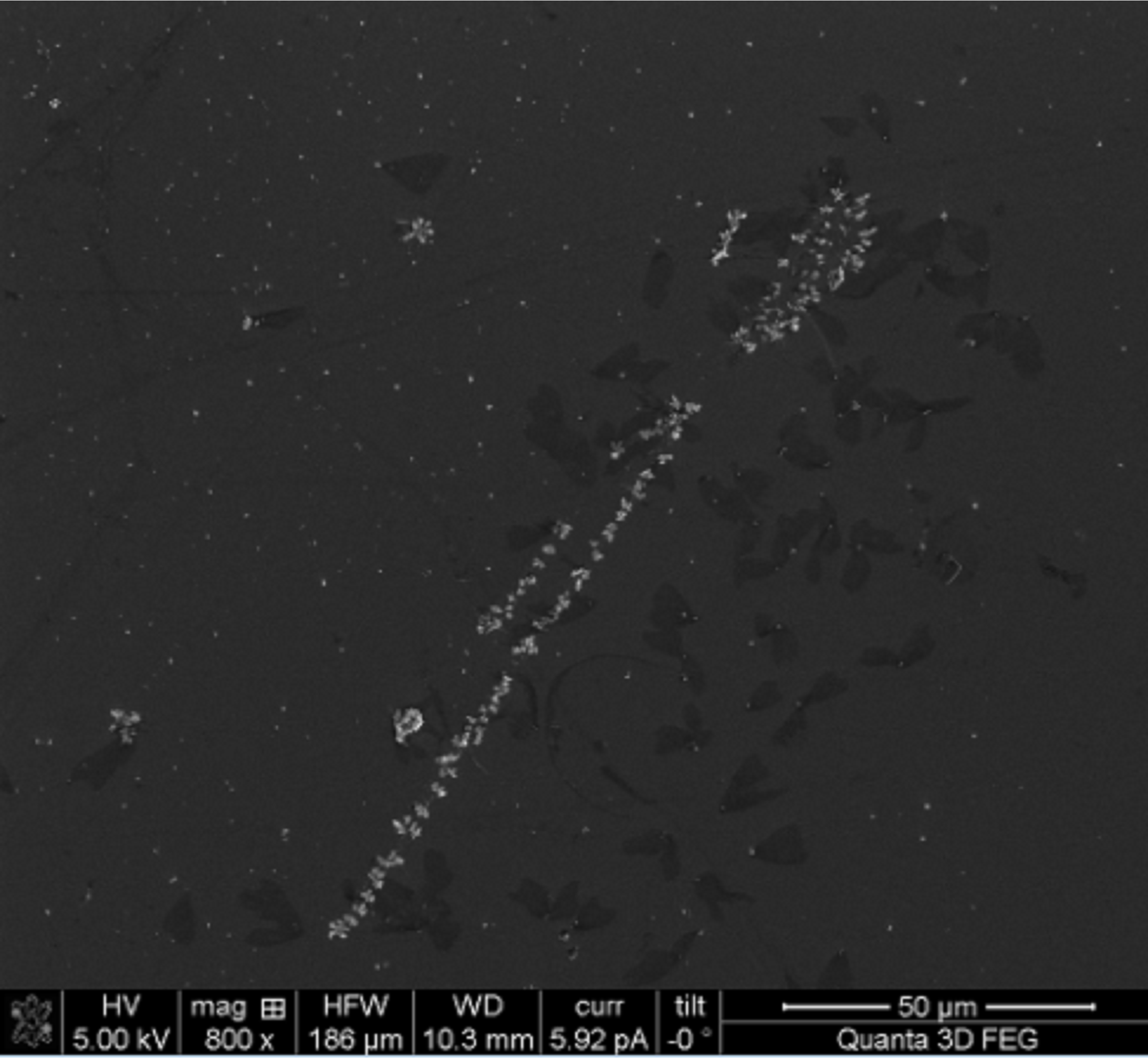

Supplement: S2 Fig — Compare with discussion in the main article. (TIF) [file pone.0195859.s002.tif]

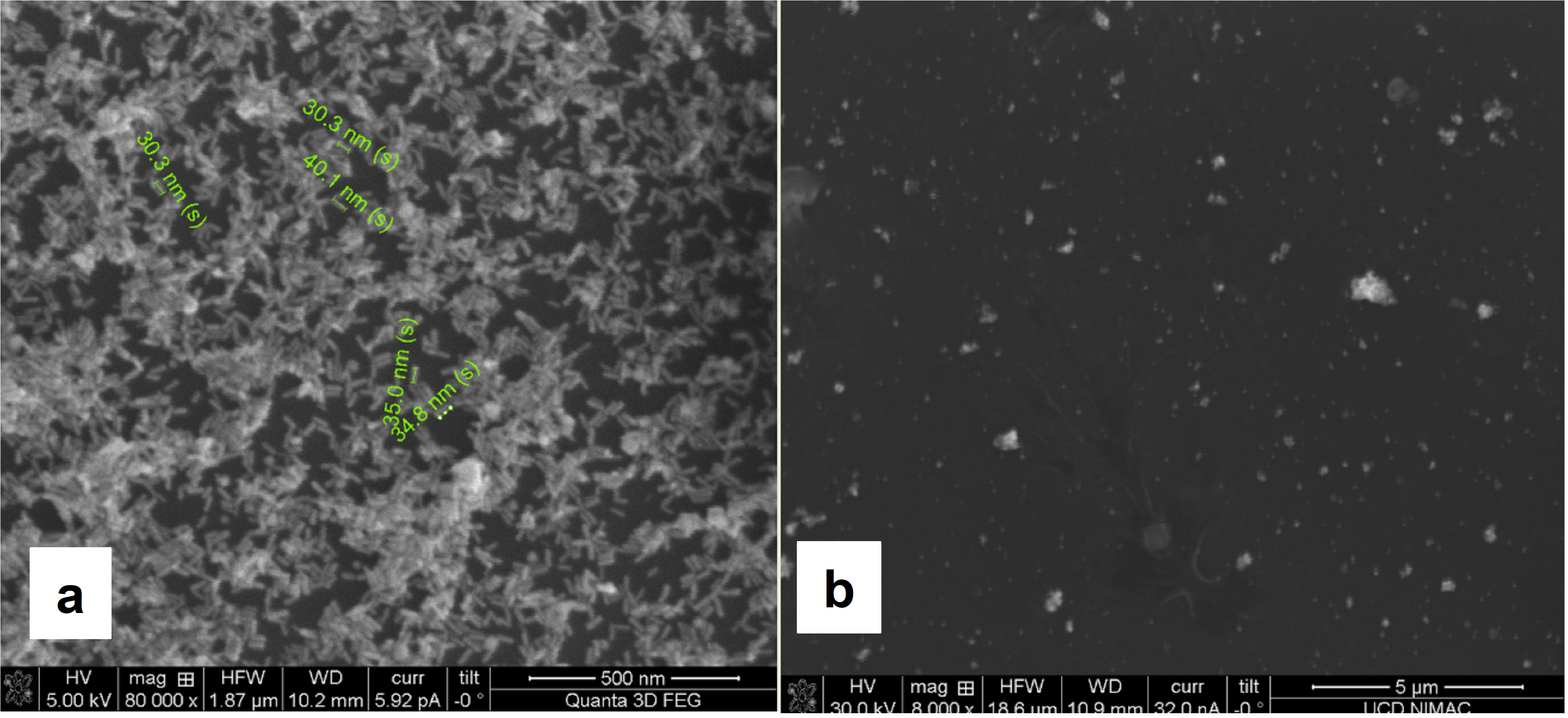

Supplement: S3 Fig — SEM analysis of a) product from grafted silicon wafer immersed in the bulk solution of gold nanorods for 24 hours; b) product from evaporation of drop-casted gold nanorods solution at 20°C. In absence of the Si-SiOx removable silicon wafer, no arrays were formed. Please note additionally the high density and uniformity (compared to the TEM analysis in the main article) when using a wider field of view (SEM, here 2 μm x 2 μm field of view). (TIF) [file pone.0195859.s003.tif]

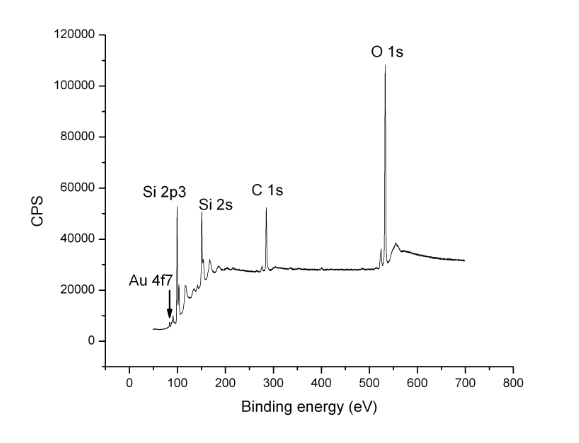

Supplement: S4 Fig — (TIF) [file pone.0195859.s004.tif]
